# Chromatin insulators: Good fences that make good neighbors

**DOI:** 10.1093/plcell/koaf157

**Published:** 2025-06-14

**Authors:** Laura Arribas-Hernández

**Affiliations:** Assistant Features Editor, Plant Cell, American Society of Plant Biologists; Instituto de Hortofruticultura Subtropical y Mediterránea La Mayora (IHSM), Consejo Superior de Investigaciones Científicas—Universidad de Málaga (CSIC-UMA), Bulevar Louis Pasteur, 49, Málaga 29010, España

Keeping distance is essential not only for people but also for genes. Within the crowded chromatin, *cis*-regulatory modules controlling the transcription of a certain gene may affect the expression of proximal loci unless insulation mechanisms are in place. Hence, chromatin insulators become essential when neighboring genes require independent expression.

Several classes of *cis*-regulatory elements have been intensively studied. These include sequences that directly promote transcription initiation (core promoters) and those that increase or decrease the initiation rate over long distances (enhancers and silencers, respectively). However, boundary sequences that isolate genes into independent transcriptional units are less well known. These so-called insulators can interrupt the action of enhancers on core promoters when located in between these 2 types of elements, but so far, very few plant insulator sequences have been proposed (Schmitz et al. 2022). In new work, Tobias Jores and colleagues identified short DNA fragments capable of blocking enhancer activity in plants (Jores et al. 2025).

Insertion of transgenes at random chromosomal locations in plant genomes has become a routine biotechnology procedure, but methods to insulate their expression from the surrounding chromatin are underdeveloped despite the obvious advantages. For example, chromatin insulators flanking transgenes could prevent the commonly observed variability among independent lines and ensure that the promoters chosen to control transgene expression are unaffected by nearby *cis*-regulatory elements responsive to unpredictable stimuli. Because the length of the few sequences with known insulator function in plants (Figure, upper left) is too large to be used in biotechnology applications, Jores et al. conducted a screen to find short DNA sequences with enhancer-blocking activity (Jores et al. 2025). They used a variant of STARR-seq (Self-Transcribing Active Regulatory Region sequencing) (Arnold et al. 2013), a technique that allowed them to identify plant enhancers in a previous study (Jores et al. 2020).

**Figure.**
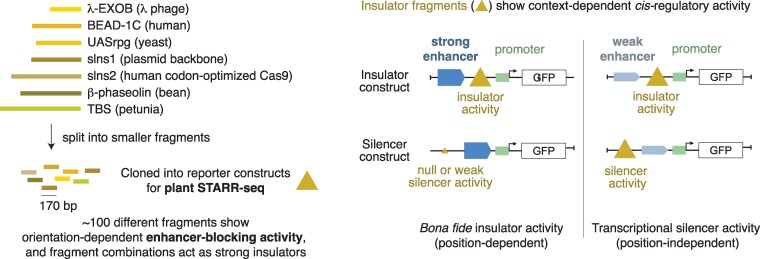
In search of short insulator sequences with activity in plants. Left, strategy followed by Jores et al. (2025) to identify short insulators within known sequences with promoter-blocking activity in plants. Right, insulators (triangles) can function as enhancer-blocking insulators and/or as silencers depending on the strength of the enhancer (arrowed blocks). Figure credit: L. Arribas-Hernández.

In search of short insulators, Jores et al. (2025) designed a reporter system consisting of a barcoded green fluorescent protein (GFP) gene whose expression is driven by 2 regulatory elements, a minimal core promoter and an upstream enhancer, separated by the insulator sequence to test. In a first approach, the authors inserted several sequences previously known to have insulator activity in plants (Figure, upper left). Upon transient expression in tobacco leaves and maize protoplasts, insertion of the insulator sequences repressed GFP expression, as expected. Then, the authors split the sequences into short (∼170 bp) overlapping fragments that were tested individually (Figure, left panel). Satisfactorily, several short fragments retained considerable insulator activity, although the variability obtained between maize and tobacco indicated some degree of species specificity (Jores et al. 2025). Although CG% generally correlated with the degree of enhancer-blocking activity, this was also orientation dependent, indicating that CG% is not the only insulation determinant (Jores et al. 2025).

To obtain increased enhancer-blocking activity, the authors ran Plant STARR-seq on combinations of 2 or 3 best-performing fragments. In this manner, the authors were able to completely block the action of the strong 35S enhancer in transient expression assays (Jores et al. 2025). Interestingly, fragments that performed well in the individual test were also good insulators when combined with other fragments, and the insulation strength was independent of whether or not the fragments belonged to the same long insulator or were copies of the same fragment. A linear model predicted that the most important position for insulator activity is next to the promoter (Jores et al. 2025).

Jores et al. tested the newly identified short insulators in different plant species, using a variety of promoter and enhancer sequences. The results showed that: 1) insulators are active in Arabidopsis, rice, and maize stable transgenic lines, although 2-mers and 3-mers were less active in these systems, and stacking additional fragments did not improve matters; 2) insulator activity is largely independent of the type of enhancer, and it can operate at long distances with complex promoters; and 3) insulator activity does not depend on the tissue or the developmental stage of the plant (Jores et al. 2025).

Finally, Jores et al. observed that insulator sequences also repressed the transcription of the GFP reporter in the absence of an enhancer. Although this silencer capacity correlated well with the insulator activity, it was more modest: up to 6% or 43% of the observed repression in the insulator construct (Figure, right) could be explained by silencer activity in tobacco and maize, respectively (Jores et al. 2025). To further investigate this phenomenon, the authors expressed insulator and silencer constructs to directly compare both capabilities and concluded that the weaker the enhancer, the higher the silencer activity of a given insulator sequence and vice versa (Figure, right). Hence, insulators and their fragments can function as enhancer-blocking insulators and/or as silencers, depending on the regulatory context.

In animals, some insulators block heterochromatin spreading, thereby preventing the inactivation of a gene by nearby epigenetic marks. However, it is not known whether plant insulators possess this activity. Something to look forward to in future work!

## Recent related articles in *The Plant Cell*

- The same authors (Jores et al. 2024) performed a comprehensive characterization of how plant enhancers integrate transcription factor activity.
- Paterson and Queitsch (2024) explored the interplay between angiosperm diversity and genome organization, with emphasis on *cis*-regulatory mechanisms.
- Yin et al. (2023) explored long-range chromatin interactions in plants.

## Data Availability

No new data were generated or analysed in support of this research.
